# Intracavernous and Intravenous Use of Contrast‐Enhanced Ultrasound in Children: A Report of Five Cases and Review of the Literature

**DOI:** 10.1002/pdi3.70038

**Published:** 2026-03-26

**Authors:** Yazi You, Juan Wang, Yuxin Tang, Zuying Li, Ruiqi Wang, Yi Tang

**Affiliations:** ^1^ Department of Ultrasound, Children's Hospital of Chongqing Medical University National Clinical Research Center for Children and Adolescents' Health and Diseases Ministry of Education Key Laboratory of Child Development and Disorders International Science and Technology Cooperation Base of Child Development and Critical Disorders Chongqing Municipal Health Commission Key Laboratory of Children's Vital Organ Development and Diseases Chongqing China; ^2^ Department of Ultrasound Medicine Renji Hospital School of Medicine Chongqing University Chongqing China

## 1 Introduction

Currently, the most commonly used enhancement imaging methods in children are contrast‐enhanced computed tomography (CECT) and dynamic contrast‐enhanced magnetic resonance imaging (DCE‐MRI). In contrast, contrast‐enhanced ultrasound (CEUS) is radiation‐free and more friendly to the pediatric population, can be performed without sedation, and is less costly and easier to perform in the clinic. The contrast agent can be injected intravenously for real‐time dynamic assessment of parenchymal perfusion or via the body cavity to enhance the imaging effect [1].

The most widely used second‐generation ultrasound contrast agent is SonoVue, which is based on sulfur hexafluoride gas and is excreted through the lungs without liver or kidney toxicity. The U.S. Food and Drug Administration (FDA) approved the use of ultrasound contrast agents in children with focal hepatic lesions and vesicoureteral reflux [2], and medical centers in Europe have published a large amount of data demonstrating the safety and efficacy of this technology, which has provided strong support to open the way for the wide application of CEUS in pediatrics [1, 2, 3, 4]. Currently, CEUS technology is mostly applied for diagnosis, differential diagnosis, and efficacy assessment of liver disease and vesicoureteral reflux in children [2, 4, 5, 6]. Our study explores the imaging of other lesions and retrospectively summarizes the experience of the nonconventional application of CEUS in five cases in children with the aim of exploring the clinical value of the nontraditional application of CEUS in children.

## 2 Information and Methods

### 2.1 Study Population

This report was prepared in accordance with the CARE (case report) guidelines recommended by the EQUATOR (Enhancing the quality and transparency of health research) [7]. Children who attended the Affiliated Children's Hospital of Chongqing Medical University between December 2021 and September 2024, all of whom had no contraindications to CEUS and were finally diagnosed by pathology, imaging, or comprehensive evaluation by clinicians, including umbilical enterocutaneous fistula combined with vesicoileal fistula, ovarian fibrofollicular meningiocytoma, renal biopsy puncture hemorrhage, testicular torsion postreplacement review, and breast fibroadenoma postablation review, were selected, one case each. We included 2 male patients and 3 female patients, aged 1.5–15.3 years. All patients had only preliminary clinical suspicion before the CEUS examination, and the final diagnosis was established by combining CEUS and pathological or further examination results.

### 2.2 Instrumentation and Methods

1. Instrumentation: Ultrasonography was performed via an ACUSON Sequoia system (Siemens Medical Solutions, USA; Mountain View, CA, USA) equipped with a 1.0–4.0‐MHz 5C1 convex array probe. All examinations were conducted by an experienced sonographer with 20 years of expertise in ultrasound.
2. Contrast agent and injection method: The contrast agent used in this study was SonoVue (Bracco, Milano, Italy). After 5 mL of 0.9% sodium chloride was mixed with the SonoVue lyophilized powder, it was injected into the subjects at a dose of 0.03 mL/kg through the elbow vein, with the maximum dose of a single injection not exceeding 2.4 mL [1], followed by a single injection of 5 mL of physiological saline. In the case of umbilical intestinal fistulas, 1 mL of microbubble suspension was injected into the lumen of the fistula through the umbilical fistula, and the contrast agent was administered with physiological saline prior to the injection. Before injection, the contrast agent was diluted with saline to ensure that its concentration was moderate and easy to inject.
3. CEUS examination method: Before the examination, the child's guardian signed an informed consent for ultrasonography. First, conventional ultrasound was used to observe the lesion, and the switch to dual‐screen display mode ensured that the surrounding normal tissues were displayed on the same surface at the same time. Then, CEUS was performed; the timer and storage button were activated, while the contrast agent was injected, and the contrast image was observed continuously and dynamically until 3–6 min after the injection of the contrast agent. Then, the dynamic image was stored on the hard disks.
4. Evaluation of contrast results: During the observation period of contrast agent injection into the body, the organ lesion was divided into two stages: early enhancement (arterial stage) and late enhancement (venous stage). The early stage of enhancement refers to the process from the beginning of perfusion of the lesion to the gradual enhancement of echoes up to the peak; the late stage of enhancement refers to the process from the beginning of the lesion's echoes decreasing to the process of decreasing to the precontrast level. The lesion was evaluated by observing the characteristics of echo enhancement of the lesion and comparing the enhancement with that of the surrounding tissues. Pathological, imaging, or clinical diagnosis was used as the gold standard to assess the application value of CEUS.

## 3 Case Reports

### 3.1 Safety

A total of five children underwent CEUS examination. The vital signs of all the children, including heart rate, respiratory rate, oxygen saturation, and blood pressure, remained stable throughout the examination and for 30 min after imaging, and there were no adverse reactions, such as nausea, vomiting, fever, or rash. To ensure the safety of the examination, all the children underwent vital sign testing as well as laboratory tests, including complete blood count and monitoring of liver and kidney function, within 24–48 h before and after the CEUS examination. These parameters were evaluated to rule out possible physiologic changes caused by the contrast medium and to ensure that the child's physical condition was not adversely affected.

**FIGURE 1:**
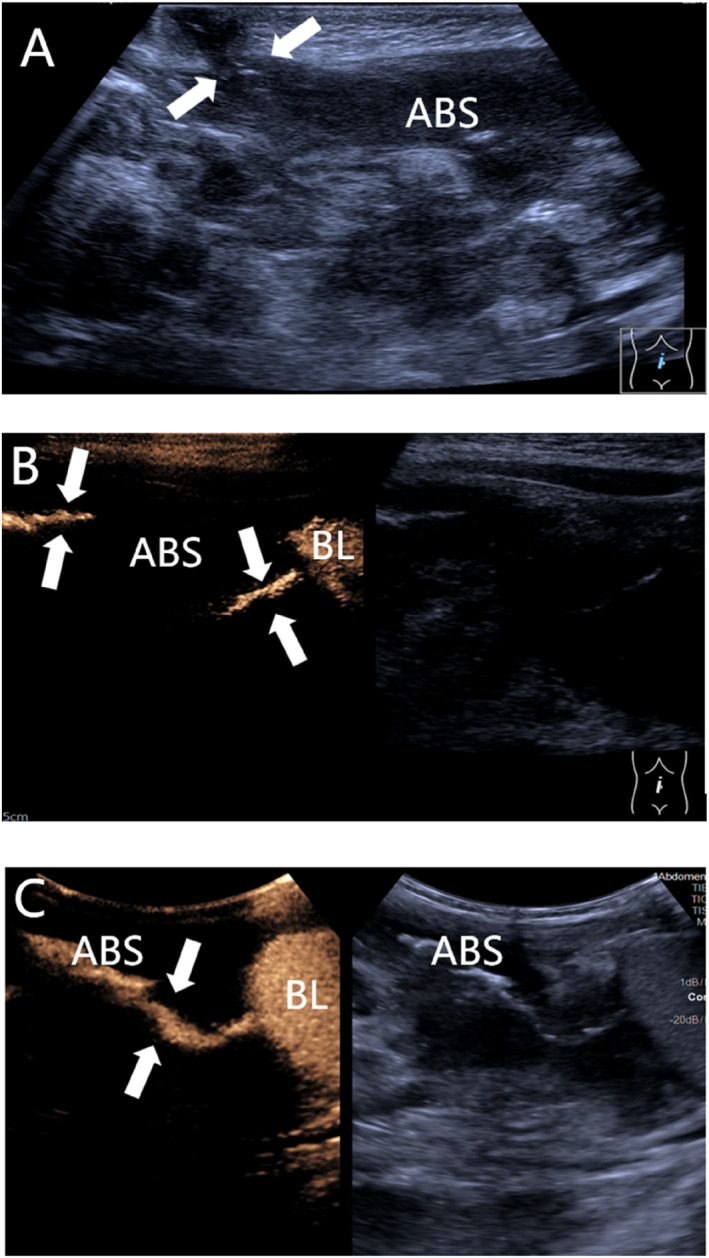
A 1.5‐year‐old male, clinically diagnosed with an umbilical intestinal fistula combined with a vesicoileal fistula. (A) Conventional ultrasound image showing an abnormal channel in the umbilicus (arrowheads) connecting to an intra‐abdominal abscess (ABS). (B) Real‐time transumbilical contrast‐enhanced ultrasound (CEUS) showing that the contrast agent reached the abdominal abscess cavity (ABS) through the fistula (arrowheads), and at the same time, the bladder (BL) connected to the abdominal abscess cavity (ABS) via a fistula (arrowheads). (C) CEUS via the bladder showing that the contrast agent reached the abdominal abscess cavity (ABS) via a fistula (arrow).

**FIGURE 2:**
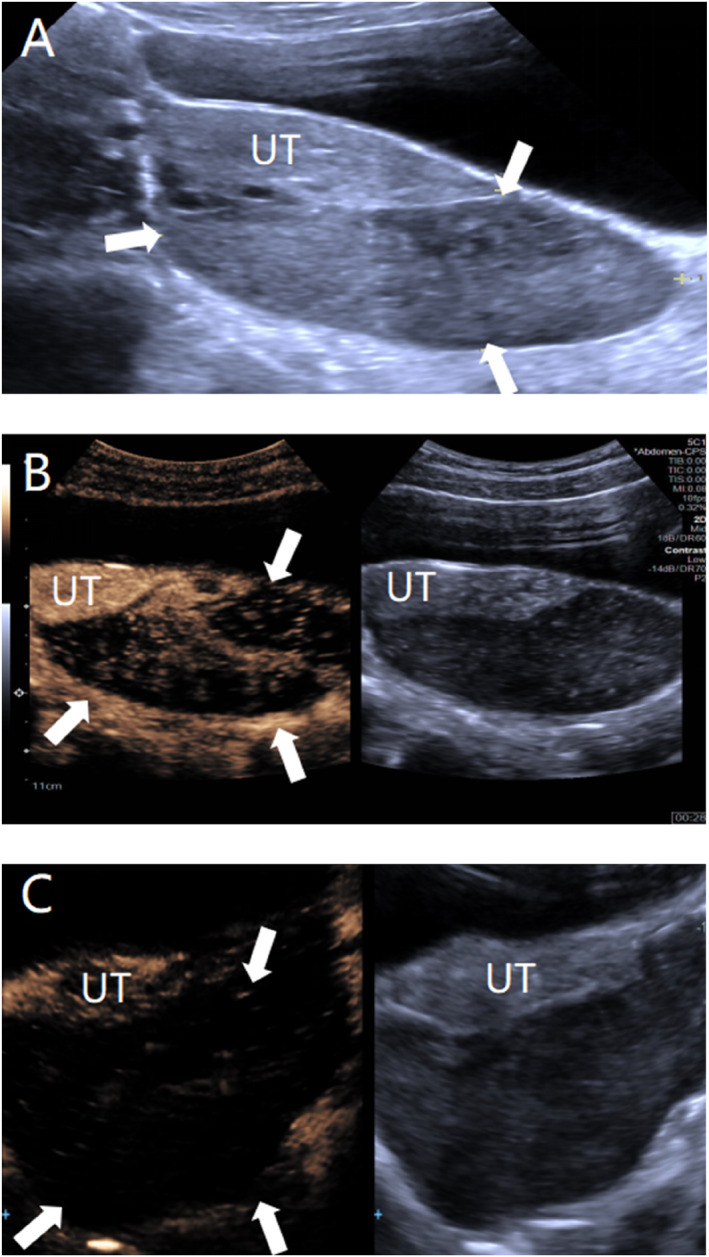
A 13.5‐year‐old female, pathologically diagnosed with left ovarian fibrous follicular membranous cell tumor. (A) Conventional ultrasound revealed a heterogeneous moderate echogenic structure from the left adnexal region to the uterus posteriorly (UT), with a follicular structure visible at the edge and abundant blood flow signals on CDFI (color Doppler flow imaging) (arrowheads). (B) Contrast‐enhanced ultrasound (CEUS) revealed that the contrast agent entered the target lesion at 16 s during the imaging process, with dendritic isoenhancement and a small amount of hypoenhancement from the outside to the inside (arrowheads). (C) Late‐stage CEUS of the lesion (arrowheads), with contrast agent fading.

**FIGURE 3:**
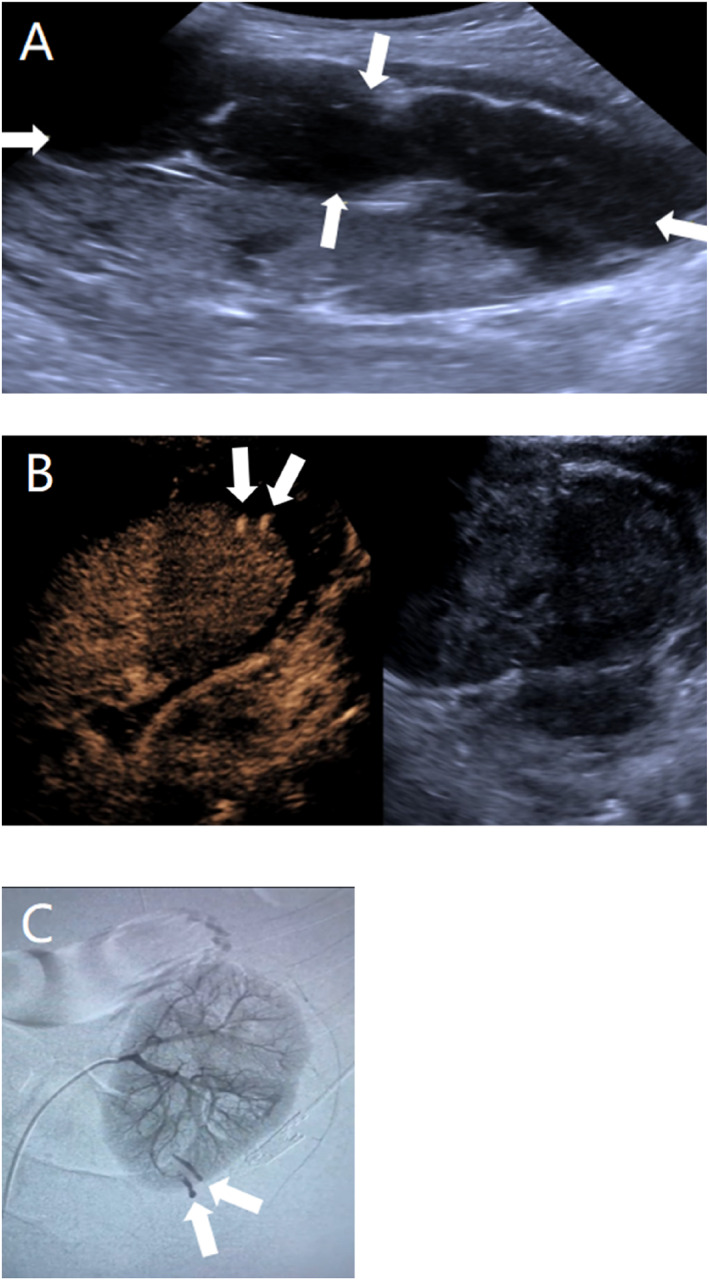
An 8.5‐year‐old female, clinically diagnosed with antineutrophil cytoplasmic antibody (ANCA)‐associated nephritis, 1 day after left renal puncture. (A) Conventional ultrasound image showing hypoechoic and echoless areas under the perinephric peritoneum of the left kidney. (B) Two ultrasound contrast leaks from the left posterior inferior renal branch artery (arrows). (C) Intraoperative DSA image showing that the left renal artery was well visualized and that two contrast leaks could be observed in the posterior inferior branch artery of the left kidney (arrowheads).

**FIGURE 4:**
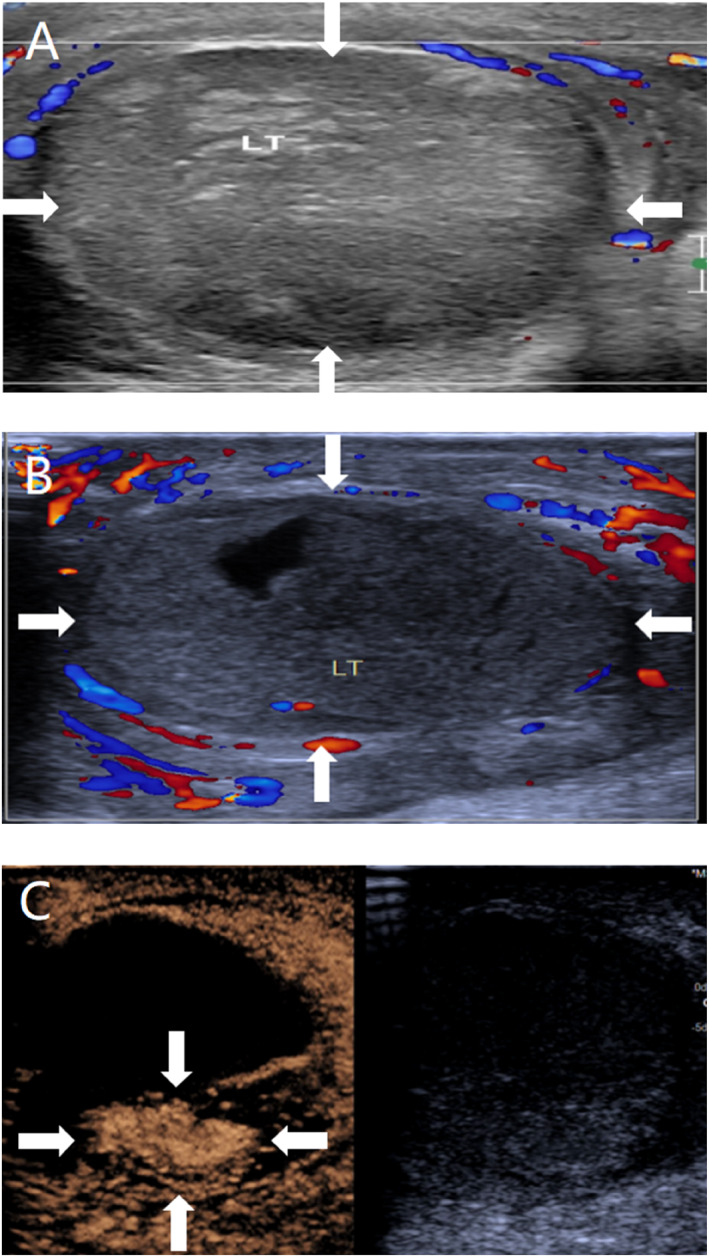
An 11.2‐year‐old male, pathologically diagnosed with necrosis of the left testis (LT) after torsion and treated with left testicular repositioning. (A) Before surgery, conventional ultrasound revealed that the axial position of the left testis had changed, with heterogeneous parenchymal echogenicity, and no blood flow signal was observed in the left testis by color Doppler flow imaging (CDFI; arrowheads). (B) After repositioning, conventional ultrasound revealed that the left testis had inhomogeneous echogenicity, with echogenic areas seen in the left testis, and no blood flow signal was observed in the center of the testis by CDFI (arrows). (C) After repositioning, most of the affected area did not experience contrast infusion, but enhancement was observed in a small area (arrows).

**FIGURE 5:**
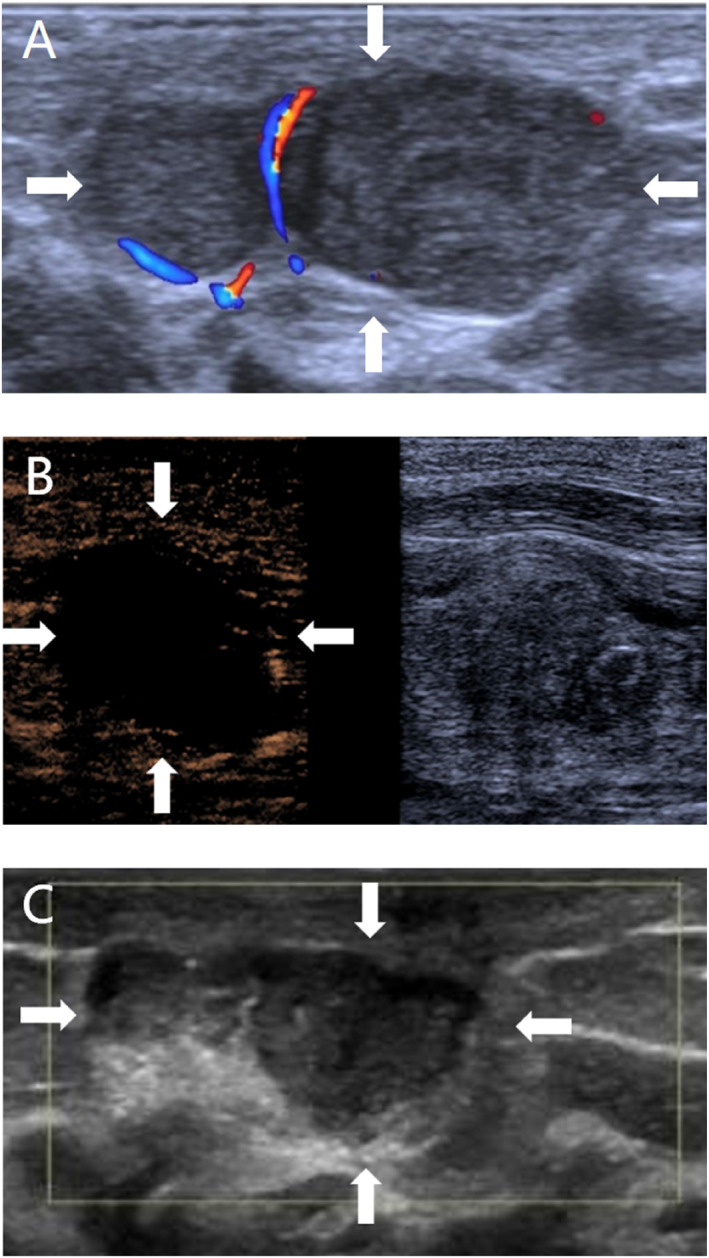
A 15.3‐year‐old female pathologically diagnosed with right breast fibroadenoma underwent radiofrequency ablation of the right breast. (A) Before ablation, routine ultrasound of the right breast revealed an inhomogeneous moderate hypoechoic lesion, and color Doppler flow imaging (CDFI) revealed a small blood flow signal within it (arrowheads). (B) One day after the ablation procedure, CEUS revealed that there was no enhancement of the ablated area throughout the entire imaging process (arrowheads). (C) Six months after ablation, routine ultrasound revealed a subhomogeneous moderate hypoechoic ablation zone, and no blood flow signal was observed within it on CDFI (arrowheads).

### 3.2 Diagnostic Efficacy of CEUS

1. Umbilical intestinal fistula combined with vesicoileal fistula: A 1.5‐year old male child was admitted to the hospital with paroxysmal abdominal pain and elevated inflammatory markers, and an ultrasound revealed an abscess cavity in the right lower abdomen. After hospitalization, the umbilicus was red, swollen, and ruptured, and the urine contained a yellowish sediment. Ultrasound revealed that the inflammatory lesion in the umbilicus was connected to the abdominal pus cavity, which was suspected to have formed an umbilical fistula after infection. X‐ray did not show typical features of intestinal perforation, and intestinal perforation was not ruled out, considering that the perforation was small and encapsulated by surrounding tissues or that the early imaging features were not obvious. Because of the presence of fecal material in the urine, the clinic considered the combination of vesicoenteric fistula and conventional ultrasound; due to the interference of intestinal gas, the depth of the fistula was not clearly visible, so tracking the fistula was difficult. Therefore, a CEUS examination was performed to clarify the situation of the fistula, and the results revealed that the contrast agent through the umbilical fistula entered the abdominal pus cavity and the bladder. Cystography revealed communication between the bladder and the pus cavity, but no communication with the umbilicus. Combined with the clinical history, these findings ruled out a patent urachus and suggested a vesicointestinal fistula, as shown in Figure 1. Surgery confirmed that the abscess cavity was located at the ileocecal perforation site on one side and connected to the umbilicus and bladder on the other side, which was consistent with the CEUS findings.
2. Ovarian fibrous follicular membranous cell tumor: A 13.5‐year‐old female patient presented with left lower abdominal pain. Routine ultrasound revealed an inhomogeneous solid mass in the left adnexal region with follicle‐like structures at the margin. Although tumor markers did not show any obvious abnormalities, imaging was needed for further evaluation due to their limited sensitivity for some ovarian tumors. A CT enhancement scan revealed that the lesion was a mixed‐density shadow with unclear boundaries, which prevented a clear determination of the nature of the mass. Given that the blood supply of different lesions is different and that CT enhancement cannot reveal the dynamic vascularization of the lesion, CEUS was performed to further clarify the diagnosis. The contrast agent showed dendritic enhancement from the outside to the inside, with equal or low enhancement in the early stage, which slowly faded in the late stage, as shown in Figure 2. These manifestations are consistent with the typical benign enhancement pattern [8], suggesting that a benign ovarian tumor was highly probable. The tumor was confirmed to be a fibrous follicular meningioma by surgical pathology.
3. Active bleeding after renal puncture biopsy: An 8.5‐year‐old female child had ANCA‐related vasculitis combined with acute renal failure. One day after renal puncture, there was a sharp drop in hemoglobin and hemorrhagic shock, and bedside ultrasound suggested the formation of a perirenal hematoma, but no clear abnormal blood flow was observed. The child's anemia worsened in the following days, and clinical consideration was given to postrenal puncture bleeding, without excluding active bleeding. Because CT and MRI contrast agents may aggravate renal damage, CEUS was performed without renal excretion of the contrast agent, and the results revealed two extravasations of contrast agent at the lower pole of the left kidney, as shown in Figure 3, suggesting active bleeding. Digital subtraction angiography (DSA) was subsequently performed to confirm the above CEUS findings.
4. Testicular torsion restoration surgery: This case involved an 11.2‐year‐old male child who was pathologically diagnosed with left testicular torsion necrosis. Preoperative routine ultrasound revealed that the axial position of the left testis had changed, and there was no blood flow signal, suggesting the possibility of testicular torsion. Postoperative routine ultrasound revealed that there was still no obvious blood supply in the affected testis, suggesting that the blood supply of the lesion was not well restored after surgery. To further determine the postoperative blood supply of the left testis, CEUS was performed on the child and revealed that most areas of the affected testis were nonperfused, with only a small portion of the area enhanced, suggesting residual activity, as shown in Figure 4. Routine ultrasound performed 9 days after surgery showed blood flow signals in the testis, indicating restoration of the testicular blood supply and preserved testicular viability. The patient remained under follow‐up.
5. Breast fibroadenoma ablation: A 15.3‐year‐old female child underwent breast radiofrequency ablation for a right breast mass, and pathology confirmed the diagnosis of breast fibroadenoma. Preoperative routine ultrasound revealed a hypoechoic lesion in the right breast with internal blood flow signals, which was considered a possible breast fibroadenoma. To clarify the blood supply of the lesion after ablation, CEUS was performed the following day, which revealed no perfusion throughout the whole process, suggesting that the lesion was ablated completely, as shown in Figure 5. Six months after the operation, a routine ultrasound revealed that a hypoechoic nodule had formed in the original ablated area, and no blood flow signals were detected, which indicated that the lesion had been ablated completely and that there was no recurrence.

## 4 Discussion

In this study, we conducted in‐depth research and practice on the diagnosis of umbilical enterocutaneous fistula combined with vesicoileal fistula in children, the differentiation of benign and malignant ovarian tumors, the determination of active hemorrhage after renal puncture, and the detection of the recovery of blood supply after testicular torsion restoration and after ablation of mammary fibroadenomas. We have accumulated valuable data and experience to provide references for subsequent research and clinical practice.

Definitive diagnosis of fistulae relies on imaging [9, 10], but conventional ultrasound has difficulty showing their complete alignment and relies on indirect signs to speculate that only some of the larger fistulae or superficial sinus tracts are visible. Intracavitary ultrasonography (IC‐CEUS) has been used in the imaging diagnosis of biliary tract and other cavitary diseases in children [11, 12, 13]. Similarly, IC‐CEUS injects a contrast agent into the fistula, which can visualize the distribution of the fistula and significantly improve the detection rate of pediatric intestinal fistulae. Chen et al. [14] concluded that IC‐CEUS can be used as an alternative to CT and MRI for the evaluation of pediatric intestinal fistulae, and the study by Ključevšek et al. [15] confirmed that it can effectively enhance the visualization of fistulae and surrounding structures. In our study, conventional ultrasound revealed only partial alignment of the umbilical intestinal fistulae, whereas CEUS clearly visualized all the fistulae and their anatomical relationships, improved the detection rate, and provided a more accurate imaging basis for surgical protocols.

The vascular features of ovarian tumors are crucial for identifying benign and malignant lesions [16], but conventional ultrasound is difficult to see clearly due to its limited resolution, and diagnostic sensitivity varies widely among physicians at different levels of experience [17]. In contrast, CEUS can more intuitively display the arterial structure and perfusion pattern of the tumor. Studies have shown that CEUS has advantages in identifying benign and malignant ovarian tumors. Hu et al. [17] found that malignant ovarian tumors often exhibit abundant blood flow, vascular distortion, and enhancement of more than 50% of the lesion area and that CEUS combined with conventional ultrasound can improve diagnostic efficacy. In a study of 51 ovarian tumors, Niu et al. [8] found that benign lesions presented peripheral‐to‐central enhancement, whereas malignant lesions presented center‐to‐center enhancement. In our study, the contrast features of CEUS were consistent with those of benign ovarian lesions, further supporting its feasibility in the differentiation of benign and malignant ovarian tumors in children.

CEUS has shown increased sensitivity and diagnostic efficacy in diagnosing active bleeding after renal biopsy [18]. He et al. [19] showed that CEUS could sensitively capture active bleeding after percutaneous renal puncture and could be used for dynamic follow‐up. In our study, CEUS clearly revealed contrast extravasation at the puncture point, which was consistent with the DSA results, and active bleeding was successfully diagnosed after renal puncture. In addition, Di Renzo et al. [20] showed that CEUS was able to accurately identify active hemorrhage in 46 pediatric organs, including 12 cases of renal hemorrhage. In this study, conventional ultrasound failed to detect renal puncture hemorrhage foci, whereas CEUS visually revealed that the contrast agent overflowed through the puncture point. Compared with conventional imaging methods, CEUS can not only effectively evaluate the extent of hematoma and active bleeding after puncture but also avoid radiation exposure, which improves the safety of children and health care workers.

Assessment of the testicular blood supply after torsion is crucial in determining the patient's condition and treatment decisions [21]. Conventional ultrasound suffers from limited resolution in demonstrating testicular blood supply, whereas Tenuta et al. [22] noted that CEUS has greater sensitivity and specificity in detecting testicular blood flow than conventional ultrasound does, especially in small‐volume, low‐velocity blood flow tests in children [23]. Yi et al. [24] found that CEUS can be used to quantitatively analyze the microcirculation of the testis and assess postoperative recovery. In this study, conventional ultrasound failed to accurately monitor the blood supply of the postoperative testis, whereas a slight enhancement signal around the testis was observed after CEUS, suggesting the recovery of microvascular perfusion, and follow‐up ultrasound also confirmed the survival of the testis. The results suggest that CEUS can sensitively detect microscopic blood flow within the testis that is difficult to recognize by conventional ultrasound, assisting in more accurate assessment of blood supply and reducing unnecessary testicular resection caused by diagnosis uncertainty in the early postoperative period or by equipment limitations.

Breast ablation in children is uncommon. Conventional ultrasound assesses the location of the lesion and monitors the ablation process, but it is difficult to accurately determine the distribution of blood flow. CEUS reveals the perfusion of the blood flow to the mass, which is helpful in evaluating the completeness of the ablation and provides guidance for supplemental ablation [25, 26]. There are no studies of CEUS after ablation of breast fibroadenomas in children, but relevant studies in adults have confirmed its value. Zhang et al. [27] found that CEUS can be used to assess ablation efficacy by observing the enhancement of the lesion. Zhou et al. [28] demonstrated that 97.5% (95% CI: 87.1%–99.9%) of patients with breast nodules could undergo CEUS for complete ablation, and the treatment was well tolerated by the patient, with no cases of severe epidermal burns. In this study, no enhancement signals were observed on postoperative CEUS, and no recurrence was detected during follow‐up, suggesting complete ablation, indicating that CEUS can accurately assess the efficacy of ablation of breast fibroadenomas in children, providing an effective tool for clinical evaluation.

According to the current national and international published literature, ultrasound contrast agents are relatively safe for use in children, with very few serious adverse reactions. For example, Yusuf et al. [29] retrospectively analyzed 305 children and recorded only 2 cases (0.66%) with mild adverse reactions, including 1 case (0.33%) of transient hypertension and 1 case (0.33%) of transient tachycardia, and in a study [3] that evaluated the safety of 312 children (including 600 intravenous injections of SonoVue), there were 6 cases (1.92%) of mild adverse reactions, including 3 (0.96%) cases of transient skin rashes and 3 (0.96%) cases of hypotension. In addition, for the safety of IC‐CEUS application, no adverse events related to contrast agents were reported in a study including 12,362 children [1]. In our center, a total of 874 pediatric CEUS examinations have been performed since the study was conducted, with 15 (1.72%) adverse events, including 12 (1.37%) mild adverse events, 3 (0.34%) moderate adverse events, and no occurrence of severe adverse events, which preliminarily confirms that the use of SonoVue intravenously and intracavernously in pediatric patients has a high safety profile.

## 5 Conclusion

We report five cases to demonstrate the accuracy and clinical value of the use of unconventional CEUS in children. Given that CEUS is noninvasive, nonradioactive, cost‐effective, easy, and reproducible, it provides valuable diagnostic information for clinicians. In recent years, the application of CEUS in pediatrics has been expanding and has played an important role in the diagnosis of a wide range of diseases. We hope that the introduction of the above cases will stimulate readers' interest in and understanding of CEUS technology and promote its application and development in the field of pediatric medicine; at the same time, we look forward to more practices and studies exploring new applications of CEUS in children in the future, providing more choices and possibilities for diagnosis and treatment in the field of pediatric medicine.

## Author Contributions

**Yazi You:** data curation, writing – review and editing, writing – original draft, conceptualization, methodology. **Juan Wang:** resources, methodology, conceptualization, writing – original draft. **Yuxin Tang:** conceptualization, methodology, resources. **Zuying Li:** writing – original draft, data curation, formal analysis. **Ruiqi Wang:** visualization, validation. **Yi Tang:** visualization, validation, investigation.

## Funding

The authors have nothing to report.

## Ethics Statement

All procedures performed in studies involving human participants were in accordance with the ethical standards of the Institutional Review Board of the Affiliated Children's Hospital of Chongqing Medical University (Certificate No. CHCMU‐XJS‐2019–20 and approval date 12 March 2019) and with the 1964 Helsinki Declaration and its later amendments or comparable ethical standards.

## Consent

Informed consent for research and publication were obtained from the parents/legal guardians of all the participants included in the study.

## Conflicts of Interest

The authors declare no conflicts of interest.

## Data Availability

The data that support the findings of this study are available from the corresponding author upon reasonable request.
